# The TrollLabs open hackathon dataset: Generative AI and large language models for prototyping in engineering design

**DOI:** 10.1016/j.dib.2024.110332

**Published:** 2024-03-16

**Authors:** Daniel Nygård Ege, Henrik H. Øvrebø, Vegar Stubberud, Martin F. Berg, Christer Elverum, Martin Steinert, Håvard Vestad

**Affiliations:** Norwegian University of Science and Technology, Richard Birkelands vei 2B, Trondheim 7034, Norway

**Keywords:** Generative AI, Prototyping, Engineering Design, ChatGPT

## Abstract

The TrollLabs Open dataset includes comprehensive information that offers a comparison of design practices and outcomes between human participants and Generative AI during a hackathon event. The dataset was curated through the running of a prototyping hackathon designed to assess the abilities and performance of generative AI, specifically ChatGPT, in the early stages of engineering design. This assessment involved comparing ChatGPT's performance to that of experienced engineering students in a hackathon setting, where participants competed by making a prototype that fires a NERF dart as far as possible. In this setup, all ideas, concepts, strategies, and actions undertaken by the AI-controlled team were autonomously generated by the ChatGPT, without human intervention or guidance, but implemented by two participants. Five self-directed baseline teams competed against the AI team. The dataset comprises 116 prototype entries and 433 edges (connection) that enable comparative analysis of design practices and performance between the team instructed solely by generative AI and baseline teams of experienced engineering design students. Prototypes and their attribute data were captured using Pro2booth, an online prototype capture platform running on participants' phones and computers. The dataset includes a transcript of the conversation between ChatGPT and the team responsible for implementing its recommendations, featuring 97 exchanges of prompts and responses. It contains the initial prompt used to instruct the AI, the objective and rules of the hackathon and the objective performance of teams, showing the ChatGPT team finishing 2^nd^ among six teams. To the authors' knowledge, the TrollLabs Open dataset is the first and only open resource that directly compares the performance of generative AI with human teams in an engineering design context. Thus, it is intended to be a valuable resource to design researchers, engineering and design students, educators, and industry professionals seeking to find strategies for implementing generative AI tools in their design processes. By offering a comprehensive data collection, the dataset enables external researchers to conduct in-depth analyses that could highlight the practical implications of integrating generative AI in design practices, possibly providing an overview of its limitations and presenting recommendations for improved integration in the design process.

Specifications Table

**Table:** 

Subject	Mechanical Engineering
Specific subject area	Using generative artificial intelligence in early-stage prototyping.
Data format	Raw, Analyzed
Type of data	Table, Text, images
Data collection	Data were collected during a prototyping hackathon explicitly designed to assess the capabilities and performance of generative AI, particularly ChatGPT, during the early stages of engineering design. The assessment involved comparing the design process and performance in a hackathon setting, where the solutions generated by ChatGPT were evaluated against those developed by experienced engineering students. In this study, ChatGPT actively participated by collaborating with two first-year PhD students, who executed the AI's design suggestions. The research utilized an online platform called Pro2booth, which is designed to track prototyping activities and prototypes throughout the design process. Participants used Pro2Booth to document their prototyping activities and design rationales through the program's online form. Demographic information was collected using MS Forms. Additionally, the chat log from the interactions with ChatGPT was downloaded and saved as a .txt file.
Data source location	• Institution: Norwegian University of Science and Technology • City/Town/Region:Trondheim, Trøndelag • Country: Norway
Data accessibility	Repository name: zenodo.org Data identification number: 10.5281/zenodo.10495102 Direct URL to data: https://zenodo.org/record/10495102

## 1 Value of the Data

• The dataset provides valuable data that could be analysed to generate insights into how generative AI, specifically ChatGPT, can be utilized in the early stages of engineering design. This is particularly important for understanding AI's potential in ideation, conceptualization, and initial problem-solving phases to exploit its benefits and mitigate its limitations.
• By offering a comprehensive collection of data on all parts of the design practices conducted by participants, the dataset enables external researchers to conduct in-depth analyses that could highlight practical implications of integrating generative AI in design practices, possibly provide an overview of its limitations and present recommendations for improved integration in the design process.
• The dataset includes the final outputs and the entire history and rationale behind design decisions that could be used to understand both AI and human-driven design decisions and their comparison.
• To the authors' knowledge, the TrollLabs Open dataset is the first open resource that directly compares the performance of ChatGPT with human teams in an engineering design context.

## 2 Background

Artificial intelligence (AI) and natural language processing (NLP) have gained increased attention due to the availability of free and easy to use tools such as ChatGPT and similar prompt-based generative AI systems. AI has transcended its traditional role as a computational aid and will likely influence how design engineers conceptualize, create, refine, and design prototypes. Although generative AI can assist in the conceptual stages of design [1], it may be inferior to tools tailored explicitly for purposes that require exacting standards and intricate technicalities [2,3]. ChatGPT, for instance, excels at text generation but has been shown to exhibit drawbacks such as forgotten information, partial responses, and a lack of output diversity for design tasks [4]. It is, therefore, uncertain how well ChatGPT performs when tasked with generating concepts and building instructions for prototypes and physical solutions that often require domain-specific knowledge at a great degree of accuracy and detail [5].

## 3 Data Description

Table 1 presents an overview of the dataset [12], detailing each file along with their respective descriptions to explain each entry and its origin.

**Table 1:** File descriptions.

Name	Description
Readme.txt	Contains information about the dataset, its curation and structure
Rules.txt	A text document including the information given to participants at the beginning of TrollLabs Open, with the design brief and objective, expected and required deliverables, constraints and assessment criteria
Prompt.txt	Initial ChatGPT prompt
Prototypes.csv	Contains the attribute data of each prototype in the database (team number, ID, name, description, domain, rationale for creation, purpose, Insights, date of capture, Method/machinery used, time to create)
Teams.csv	Contains the role of each team, regular participants denoted by the name “Participant” or controlled by ChatGPT denoted by “ChatGPT.”
Test_results.csv	Contains results from the final performance test of designs after the hackathon
Chat.txt	Copy of the chat between participants and ChatGPT. Prompts have the header “participant” and answer the header “ChatGPT.”
Demographics.csv	Demographics of participants, including background and relevant experience.
Media.zip	Contains media files uploaded alongside prototypes in Pro2booth with references to corresponding prototypes in the Prototypes.csv file.

## 4 Experimental Design, Materials and Methods

### 4.1 TrollLabs Open Hackathon

The TrollLabs Open hackathon was run to elucidate the capabilities and performance of generative AI, specifically ChatGPT, in the early stages of engineering design by comparative analysis in a hackathon setting, where its outputs and solutions were evaluated against those developed by experienced engineering students [6]. ChatGPT actively participated in the hackathon by collaborating with two first-year PhD students, who executed the AI's design suggestions.

The 48-hour challenge involved developing a prototype capable of launching a foam NERF dart the farthest distance under specific constraints in a university makerspace. It required the creation of a free-standing prototype without external power or air sources. Performance was measured based on the distance a NERF dart was launched, and teams were limited to one counting test on the final day of the challenge. These results are provided in the Test_results.csv file. Participants, including ChatGPT's team, were given identical conditions and resources. The complete list of rules and requirements is provided in the rules.txt file. The design task and rules were given to the participants at the start of the challenge.

ChatGPT was given a specific prompt outlining its role in the hackathon as the sole decision-maker for its team. The prompt is provided in the prompt.txt file. The human participants acted as executors of ChatGPT's instructions, with a mandate to seek clarification and provide feedback but not to influence the decision-making process. This setup aimed to evaluate ChatGPT's autonomous problem-solving and decision-making capabilities within the confines of the engineering design challenge. ChatGPT-4.0 (Oct. 19^th^, 2023) was chosen as the best-fitting language model because of its analytical skills and refined understanding [7], as well as being the most recognized LLM available to the AI-controlled team during the study. The full chat is provided in the Chat.txt file.

Ten voluntary participants were recruited for the study alongside the ChatGPT team. These individuals were selectively invited to participate due to their active involvement in writing their master's thesis at TrollLabs, the research group in which this study has been carried out. This ensured their relevant expertise and interest in the field of early-phase engineering design and competitiveness in the hackathon. Masters’ students were unaware that ChatGPT was instructing one team to mitigate biased behaviours. Complete individual demographics are provided in the Demographics.csv file, detailing backgrounds and relevant experience. Demographics were captured using an online form.

### 4.2 Data Capture Method – Pro2booth

Pro2booth is an online prototype capture system designed to capture design activities by logging prototypes and their attributes [8]. Pro2booth have previously been used to capture large prototype datasets in similar events [[9], [10]], and to provide an accurate representation of the users' design process [11]. This merited the reuse of Pro2booth for this study. The hackathon rules encourage using the platform by rewarding points for uploading prototypes. All attribute fields had to be filled for acceptance into the database to ensure prototypes were captured correctly. Participants interacted with Pro2booth through a website where they could upload prototypes and attributes in a form consisting of free-text entries and drop-down menus. Users, prototypes, and projects make up the nodes and edges of the dataset, all of which are linked through a graph database. Pro2booth captures the following attributes for each prototype: an ID for reference, a name, a team number, description (free text), domain (drop-down menu), media (picture, video, CAD files), who created it, Influences, rationale (free text), purpose of creation (drop-down menu), Insights (free text), date of capture, method/machine used (drop-down menu), time to create (drop-down menu). Prior entries were editable until the completion of the hackathon. Upon conclusion of the hackathon, the data from Pro2booth was consolidated into a .json file. This file was then parsed to generate CSV files, which were categorized to extract prototypes, users and projects. This process resulted in creating the 'prototypes.csv' file in the dataset. This file encapsulates detailed information about each prototype, including its various attributes and the team responsible for its creation. Media content was required alongside prototype uploads, all referenced in the ‘prototypes.csv ´ and saved in the media.zip file. Additionally, a 'Teams.csv' file lists each team number and identifies their role in the hackathon. The roles are categorized as either “participant” for human teams or “ChatGPT” for the team directed by the generative AI, clearly differentiating the teams based on their operational dynamics.

## Limitations

The data might be influenced by the human involvement necessary for executing ChatGPT's ideas, with the potential for either excessive or insufficient human input affecting the practicality and integrity of the outcomes. The dataset also faces limitations due to the subjective nature of interpreting ChatGPT's instructions, which were sometimes vague. This could lead to variability in the execution of instruction, potentially affecting the consistency and replicability of the data.

## Ethics Statement

All participants involved in the TrollLabs Open Hackathon provided written informed consent, confirming that they had read and understood the consent form, ensuring that they were fully aware of the nature of the study, its objectives, and their roles within it.

## CRediT authorship contribution statement

**Daniel Nygård Ege:** Conceptualization, Methodology, Validation, Investigation, Data curation, Writing – original draft, Writing – review & editing, Project administration. **Henrik H. Øvrebø:** Conceptualization, Investigation, Data curation, Writing – review & editing. **Vegar Stubberud:** Conceptualization, Investigation, Data curation, Writing – review & editing. **Martin F. Berg:** Conceptualization, Investigation, Data curation, Writing – review & editing, Project administration. **Christer Elverum:** Conceptualization, Writing – review & editing. **Martin Steinert:** Conceptualization, Methodology, Writing – review & editing, Supervision. **Håvard Vestad:** Conceptualization, Methodology, Investigation, Data curation, Writing – original draft, Writing – review & editing, Supervision, Project administration.

## Data Availability

TrollLabs Open Dataset (Original data) (Zenodo).
